# Screening anticancer peptides performance in organotypic prostate tumor‐stroma 3D models

**DOI:** 10.1002/ijc.70333

**Published:** 2026-01-13

**Authors:** Bárbara Matos, Maria V. Monteiro, Matilde R. Lagarto, John Howl, Carmen Jerónimo, Vítor M. Gaspar, João F. Mano, Margarida Fardilha

**Affiliations:** ^1^ Laboratory of Signal Transduction, Department of Medical Sciences, Institute of Biomedicine—iBiMED University of Aveiro Aveiro Portugal; ^2^ Cancer Biology and Epigenetics Group, IPO Porto Research Center (CI‐IPOP) Portuguese Institute of Oncology of Porto (IPO Porto) Porto Portugal; ^3^ Department of Chemistry, CICECO University of Aveiro, Campus Universitário de Santiago Aveiro Portugal; ^4^ School of Health and Life Sciences City South Campus SCT101, Birmingham City University Edgbaston UK

**Keywords:** 3D spheroids, heterotypic spheroids, cancer treatment, peptide, prostate cancer

## Abstract

Prostate cancer (PCa) poses a significant concern in the realm of cancer, representing a continuous challenge for the scientific community to discover effective therapeutic approaches. Among emerging strategies, anticancer peptides have garnered attention for their potential to disrupt protein–protein interactions. Targeting protein phosphatase 1 (PP1) complexes through PP1‐disrupting peptides holds promise for selectively impeding critical pathways in the development and progression of cancer. In this context, CAVPENET peptide was designed to specifically target and disrupt the complex formed between PP1 and caveolin‐1, a contributor to the progression of PCa. Previous research has revealed that CAVPENET inhibits the growth of PCa cell 2D monolayers, primarily by modulating PP1 activity. In this study, we developed an increasing physiomimetic human 3D PCa/prostate cancer‐associated fibroblast heterotypic spheroid model to evaluate the tumor‐suppressive activity of CAVPENET peptide in a more relevant preclinical context. Our findings reveal the formation of morphologically well‐defined tumor microtissues that increase their size and cellular density over time, characteristics of *in vivo* tumors. Upon incubation with CAVPENET, PCa spheroids exhibited decreased growth and viability. In contrast, CAVPENET treatment (20 μM) did not influence CAFs monotypic spheroids growth. In conclusion, our results underscore the relevance of employing 3D PCa‐stroma heterotypic models for evaluating anticancer therapeutics and emphasize the therapeutic potential of CAVPENET peptide for PCa.

## Graphical abstract

What's new?

Peptide‐based drugs have emerged as promising anticancer therapies for their potential to disrupt protein–protein interactions. In this study, the authors developed a physiomimetic human 3D prostate cancer spheroid model to assess the efficacy of the CAVPENET peptide in inhibiting tumor growth. Upon incubation with CAVPENET, prostate cancer spheroids exhibited decreased growth and viability, consistent with previous results in conventional 2D monolayer prostate cancer cell cultures. The findings reinforce the potential of CAVPENET as an innovative anticancer therapy for prostate cancer and underscore the enhanced suitability of 3D spheroid models for evaluating anticancer peptides at the preclinical level.

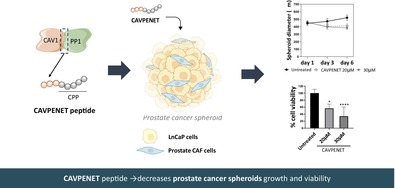

**Abbreviations**
CAV‐1: caveolin‐1
PCa: prostate cancer
PCAF: prostate cancer‐associated fibroblast
PI: propidium iodide
PP1: protein phosphatase 1
TAMRA: 6‐carboxyl‐tetramethylrhodamine
ULA: ultra‐low adhesion

## 1 INTRODUCTION

Prostate cancer (PCa) is one of the most prevalent types of cancer and poses a clinical challenge, particularly in advanced stages.^1^ The development of an effective therapeutic approach for this disease continues to engage the scientific community. In this context, the protein phosphatase 1 (PP1) has emerged as a promising drug target in different types of cancer due to its involvement in tumor‐related cellular processes (e.g., proliferation and metastasis).^2^ Nevertheless, the clinical potential of blocking the PP1 active site was constrained by multiple undesirable adverse effects, a consequence of diverse intracellular functions of PP1. More recently, the concept of target‐specific PP1 complexes has spurred the discovery and synthesis of selective modulators of PP1 activity.^3^

Over the years, several small molecules have been developed to target different PP1 complexes.^4^ In particular in PCa, the literature describes a number of small molecule compounds designed to disrupt the interaction of PP1 with histone deacetylases.^5^, ^6^ Recently, peptide‐based drugs are gaining momentum as anticancer therapeutics.^7^ In contrast to small molecule, PP1‐disrupting peptides provide several advantages, including higher selectivity and potency, reduced immunogenicity, and enhanced safety.^8^ Indeed, the development of PP1‐disrupting peptides is highly attractive and relatively underexplored in comparison to other approaches. To the best of our knowledge, to date, only three peptides designed to target PP1/GADD34^9^, ^10^ or PP1/CDCA2^11^ complexes, exhibit anticancer activity in *in vitro* preclinical studies. The effect of the peptide developed by Obeid et al.^12^ was also evaluated *in vivo* using a mouse xenograft model and a reduction in tumor growth was observed. In this framework, recently, our team designed and synthesized a bioactive cell‐penetrating peptide—CAVPENET, to selectively target and interfere with the interaction between PP1 and caveolin‐1 (CAV1), which has been implicated in PCa progression.^12^ The CAVPENET peptide sequence also includes penetratin, which plays a key role in its penetration into solid tumors. This peptide showed anticancer potential in LnCaP and PC‐3 cell lines, by decreasing cancer cells survival and invasiveness. The modulation of PP1 activity and consequent suppression of AKT signaling, inducing alterations in the metabolism of PCa cells, was proposed as the primary mechanism involved in CAVPENET anticancer activity.^13^

Previous *in vitro* pharmacodynamic studies of the CAVPENET peptide were performed with common 2D monolayer cell cultures. Clearly, these approaches poorly mimic the tumor architecture and complex multicellular microenvironment.^14^ As a result of the demand for more suitable cellular models of human disease, 3D cultures have received increased attention over the past several years.^15^ As compared to flat cultures, 3D models mimic more accurately critical *in vivo* tumor hallmarks, including cell–cell contacts, cellular morphology and organization, oxygen/nutrient gradients, gene expression and resistance to therapies.^16^ Most pertinently, 3D *in vitro* PCa cultures have shown to be more resistant to docetaxel and anti‐androgen treatment than corresponding 2D cultures.^17^, ^18^ Among 3D culture models, spheroids have been gaining significant attention in oncology. Their cost‐efficient features, along with the ability to co‐culture cancer cells with tumor stroma cells in a reproducible mode, while mimicking the tumor bio architecture, render the spheroid model particularly suitable to test anticancer therapeutics at a preclinical level.^15^ Including stromal components in 3D *in vitro* models is highly valuable considering that the tumor microenvironment plays a crucial role in the development, progression, resistance to therapeutics and metastasis. Cancer‐associated fibroblasts (CAFs) are vital elements of the tumor microenvironment and active participants in PCa progression and metastasis. A major role in altering PCa cells’ metabolism and decreasing its sensitivity to drugs was also described for prostate CAFs (PCAFs), although the molecular mechanisms are not fully understood.^19^ For instance, human PCAFs inhibited the effectiveness of docetaxel^20^ and doxorubicin^21^ in prostate tumors, highlighting the importance of heterotypic spheroid models in anticancer drug testing.

Gathering on this, herein we developed an organotypic 3D *in vitro* model of PCa by co‐culturing LnCaP with PCAF cells. The generated model was used for assessing the putative anticancer properties of CAVPENET peptide and a scrambled homologue (CAVPENET control). Previous investigations in 2D monolayer PCa cell cultures revealed that CAVPENET effectively reduces both the viability and invasive ability of PCa cells. Notably, in the highly biomimetic 3D PCa model we developed, the peptide maintained its tumor suppressor activity, exhibiting comparable effects on cell viability. Altogether, our findings reinforce the potential of CAVPENET as an innovative anticancer therapy for PCa.

## 2 MATERIALS AND METHODS

### 2.1 Peptides design and synthesis

The peptides used in the present study were prepared as described in Matos et al, 2024.^13^ Briefly, CAVPENET peptide was designed based on the peptide sequence of the PP1‐binding motif of CAV1, which was coupled to the cell penetrating peptide penetratin. A scrambled sequence, to be used as control was also designed – CAVPENET control (Table 1).

**TABLE 1:** Designation, sequence and molecular masses of the peptides used in this study.

Peptides designation	Sequence	Mass (g/mol)
CAVPENET	VV**KIDF**EDRQIKIWFQNRRMKWKK	3191.84
CAVPENET control	VV**DFIK**EDRQIKIWFQNRRMKWKK	3191.84

*Note*: The PP1 binding motif is in bold, and the cell penetrating peptide (penetratin) is underlined. Both peptides were synthesized as C‐terminal amides.

These peptides were synthesized by microwave‐assisted solid phase peptide synthesis and purified by semipreparative scale high‐performance liquid chromatography.^13^ Fluorescent analogues of the peptides were also synthesized by aminoterminal acylation with 6‐carboxyl‐tetramethylrhodamine (TAMRA)—TAMRA‐labeled peptides.

### 2.2 Cell culture

LnCaP (RRID: CVCL_0395; ATCC‐CRL‐1740) androgen‐dependent prostate cancer cells were cultured in Roswell Park Memorial Institute media‐1640 with L‐glutamine (RPMI‐1640, Gibco, Life Technologies, USA), supplemented with 10% (v/v) fetal bovine serum (FBS, Gibco, Life Technologies, USA) and 1% (v/v) penicillin/streptomycin mixture (Gibco, Life Technologies, USA). Immortalized human prostate cancer‐associated fibroblasts (PCAF) (ATCC‐CRL‐3290) were cultured in EMEM medium supplemented with 10% FBS and 1% antibiotics mixture (penicillin/streptomycin). Both cancer and stromal cells were maintained in an incubator (MCO‐170AICUV, Panasonic, Germany) at 37°C, with a 5% CO_2_ atmosphere under fully humidified conditions. All human cell lines have been authenticated using STR profiling within the last 3 years. All experiments were performed with mycoplasma‐free cells.

### 2.3 Spheroid cultures

**FIGURE 1:**
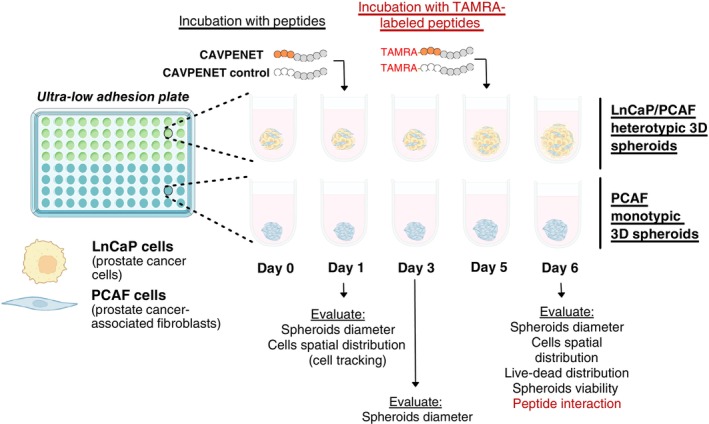
Schematic representation of prostate cancer 3D spheroids assembly, including the incubation with the peptides and the dose regime.

For generating 3D monotypic PCAF spheroids, cells were plated into 96‐well ultra‐low adhesion (ULA) U‐bottom 96‐well plates (10,000 cells per well, Corning). For heterotypic LnCaP/PCAF cultures, tumor cells and CAFs were plated at a ratio of 3:7 (3000 LnCaP cells + 7000 PCAF cells), consistent with the observed *in vivo* aggressive tumors,^22^ in ULA plates. Spheroid cultures were maintained in RPMI‐1640 medium supplemented as described above. The diameter of spheroids was determined at day 1, 3, and 6 for all the experimental conditions (*n* = 5 spheroids/experimental condition in three independent experiments) (Figure 1).

### 2.4 Microscopic evaluation of cellular uptake

Monotypic PCAF and heterotypic LnCaP/PCAF spheroids were plated in ULA plates at the described cellular densities and spheroids were allowed to form for 5 days. After this time, the spheroids were incubated with 20 and 30 μM of TAMRA‐conjugated CAVPENET control and CAVPENET peptides for 24 h at 37°C and 5% CO_2_ atmosphere. Following the incubation time, the culture medium was removed, and the spheroids were washed 3× with PBS1x (pH = 7.4). Subsequently, the spheroids were gently washed in RPMI‐1640 medium without phenol red and imaged. The models were observed by using a widefield fluorescence microscope (Axio Imager M2, Carl Zeiss, Germany) equipped with a 3.0 MPix monochromatic camera. Untreated spheroids were used as background reading. These experiments were performed in triplicate.

### 2.5 Live‐dead assay

Monotypic PCAF and heterotypic LnCaP/PCAF spheroids were plated in ULA plates, as described above. After maturation at 37°C, 5% CO_2_, for 24 h, the spheroids were incubated with 20 μM of CAVPENET control and CAVPENET. After 5 days of incubation with the peptides, a live/dead cell assay was performed (Figure 1). In brief, monotypic and heterotypic spheroids were incubated with Calcein AM (Life Technologies, Thermo Fisher Scientific, USA) and propidium iodide (PI) (Thermo Fisher Scientific, USA), both at a concentration of 5 μg/mL, in FBS‐free culture medium at 37°C, 5%CO_2_, for 30 min and then washed with PBS and observed under a widefield fluorescence microscope (Axio Imager M2, Carl Zeiss, Germany).

### 2.6 Cell viability assay

Spheroid PCAF and LnCaP/PCAF cultures were plated in ULA plates at the cellular densities described above and after 24 h were incubated with 20 and 30 μM of CAVPENET control and CAVPENET peptides. Untreated cells were also included in the assay and used as control (considered 100% viability). After 5 days of incubation with the peptides at 37°C in a 5%CO_2_ atmosphere, cell viability was determined by using the CellTiter‐Glo® 3D Cell Viability Assay (Promega, Madison, WI, USA). After removing the culture medium, a mixture of medium and cell viability reagent (1:2) was added to each well and incubated for 30 min, at RT (the first 5 min with agitation). After the incubation time, 100 μL of the mixture from each well were transferred to a white‐bottom 96‐well plate and the luminescence was read in the microplate reader Tecan Infinite® 200 PROseries, Mannedorf, Switzerland. Three to five replicates of each condition were analyzed in three independent experiments.

### 2.7 Cell tracking analysis

Prior to the formation of spheroids, cells were incubated with cell tracking lipophilic dyes: PCAF cells with DiD and LnCaP cells with DIO (2 μM per 1 × 10^6^ cells), at 37°C, for 20 min. Then, heterotypic LnCaP+PCAF spheroids were assembled, as described above. After 24 h, the spheroids were incubated with 20 μM of CAVPENET control and CAVPENET peptides. Labeled spheroids were visualized in a widefield fluorescence microscope (Axio Imager M2, Carl Zeiss, Germany) at predetermined time‐points (Figure 1).

### 2.8 Statistical analysis

Statistical analysis was performed using GraphPad Prism version 8.2.1 (GraphPad Software, USA). Descriptive data are represented as mean ± standard deviation (SD) of at least three independent experiments. Differences between the experimental groups were determined using the Kruskal–Wallis test followed by the post‐hoc Dunn's test or with two‐way ANOVA with Tukey multiple comparison test (whenever data were normally distributed). The p‐value <.05 was considered statistically significant.

## 3 RESULTS

### 3.1 Peptides interaction with prostate cancer 3D spheroids

**FIGURE 2:**
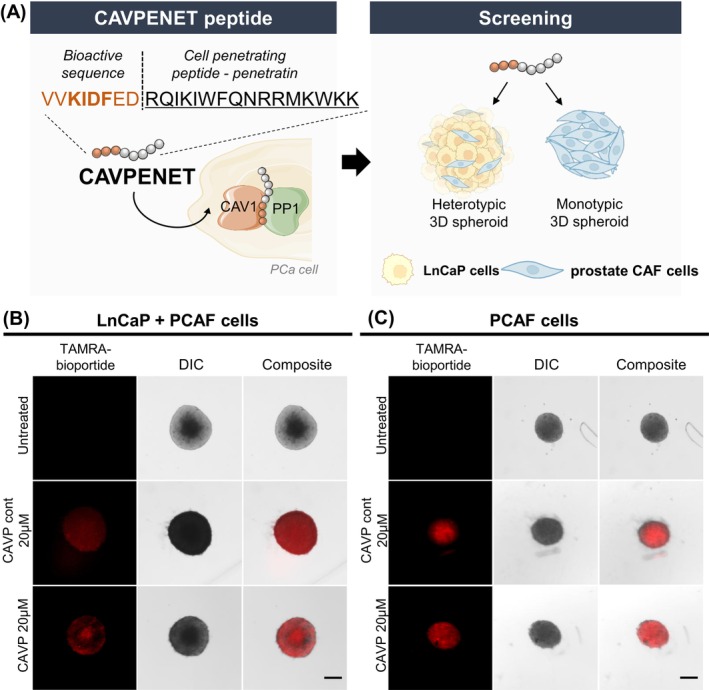
(A) Schematic representation of CAVPENET peptide design and screening in prostate cancer 3D spheroids and translocation of CAVPENET control and CAVPENET peptides into (B) LnCaP/PCAF heterotypic 3D spheroids and (C) PCAFs monotypic 3D spheroids. 3D spheroids (LnCaP+PCAF and PCAF) were incubated with TAMRA‐labeled peptides (20 μM) for 24 h at 37°C in a 5% CO_2_ atmosphere. Representative images from three replicates are represented. DIC, Differential interference contrast. Scale bar: 200 μm.

Firstly, we aimed to evaluate if the synthesized peptides were able to interact with the 3D microtumors mass *in vitro*. To confirm the interaction of the peptides with LnCaP/PCAF heterotypic spheroids, the spheroids were incubated with TAMRA‐labeled CAVPENET control and anticancer CAVPENET peptide (Figure 2A).

Both peptides were successfully able to interact with tumor‐stroma 3D spheroids after 24 h incubation, as demonstrated by the fluorescent labeling. The CAVPENET control peptide exhibited a more homogeneous distribution throughout the spheroid when compared with CAVPENET peptide (Figure 2B). The interaction of CAVPENET control and CAVPENET with spheroids only containing PCAF cells was also evaluated. As indicated (Figure 2C), both peptides interacted with 3D PCAF monotypic spheroids.

### 3.2 Effect of CAVPENET peptide in prostate cancer 3D spheroids growth

**FIGURE 3:**
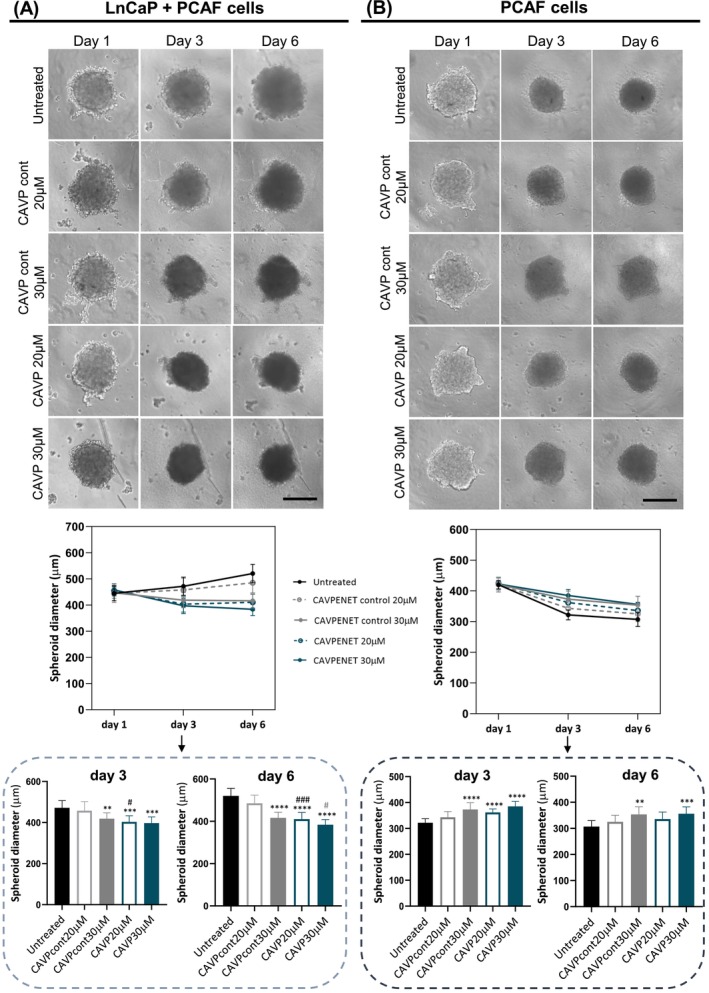
Diameter of (A) LnCaP/PCAF heterotypic 3D spheroids and (B) PCAF monotypic 3D spheroids, along the time (day 1, 3, and 6) after incubation with 20 and 30 μM of CAVPENET control and CAVPENET peptides. A representative image of each condition in different timepoints is represented in the upper panel (Scale bar: 300 μm), and the respective quantification of spheroids diameter in each timepoint in the lower panel. Data is presented as mean ± SD of three independent replicates with *n* = 5. **p <.01, ***p <.001, ****p <.0001, compared to the untreated condition; #*p* <.05, ###*p* <.001, compared to the CAVPcont20μM; #*p* <.05, compared to CAVPcont30μM.

To elucidate the anticancer potential of CAVPENET peptide, optical contrast micrographs of heterotypic LnCaP/PCAF spheroids were obtained at different time frames (i.e., at day 1, 3, and 6). Also, the diameter of spheroids was monitored during the treatment. The same methodology was followed for monotypic PCAF spheroids. Heterotypic LnCaP/PCAF spheroid images exhibited the formation of morphologically defined tumor microtissues in all experimental conditions (Figure 3A). Importantly, the untreated spheroids' diameter increased over the time‐frame, potentially indicating cellular proliferation. Conversely, the diameter of spheroids treated with CAVPENET at both concentrations exhibited a reduction in diameter. Particularly, at day 6, the diameter of spheroids incubated with 20 and 30 μM of CAVPENET was significantly decreased (*p* <.0001). The control peptide only significantly impacted spheroid growth at the higher tested concentration (*p* <.0001). Remarkably, the effect of CAVPENET was noticeably more pronounced than that of CAVPENET control at a similar concentration (*p* = .0005 for 20 μM and *p* = .0464 for 30 μM) (Figure 3A).

Conversely, untreated spheroids composed of only PCAF cells become more compact with time, decreasing their size (Figure 3B). This effect was more pronounced in the untreated spheroids. At day 6, the spheroids' diameter was significantly increased only for the highest concentrations (30 μM) of the peptides (*p* = .0014 for CAVPENET control and *p* = .0003 for CAVPENET). No significant differences were observed between the two peptides (Figure 3B).

### 3.3 Impact of anticancer peptide on prostate cancer 3D spheroids viability

**FIGURE 4:**
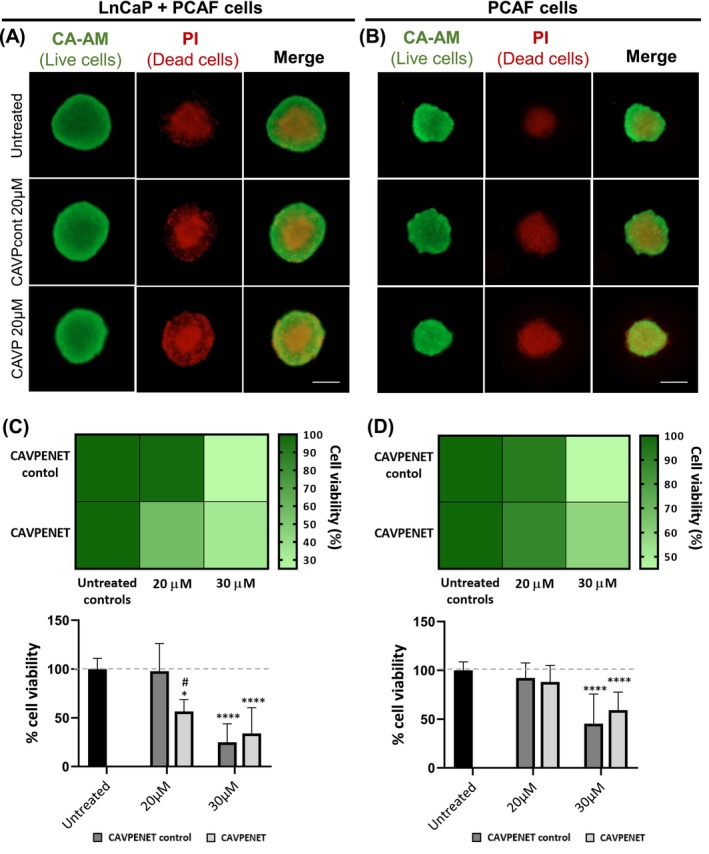
Live/dead 3D spheroids at day 6 of (A) LnCaP/PCAF heterotypic 3D spheroids, and (B) PCAF monotypic 3D spheroids after incubation with 20 μM CAVPENET control and CAVPENET peptides. Live cells were labeled with Calcein‐AM (green) and dead cells with propidium iodide (PI) (red). A representative image of each condition is represented (Scale bar: 200 μm). Spheroids viability at day 6 of (C) LnCaP/PCAF 3D spheroids and (D) PCAF 3D spheroids (including an heatmap and the statistical analysis) after incubation with 20 and 30 μM of the peptides. The percentage of viability were calculated through the ratio between treated and untreated conditions, considering untreated condition as 100% viability. Data is presented as mean ± SD of three independent replicates with *n* = 5. *p <.05, ****p <.0001, compared to the untreated condition; #*p* <.05, compared to the CAVPENET control 20 μM.

To determine whether CAVPENET peptides impacted the viability of spheroid cells, both Calcein‐AM (live)/propidium iodide (PI) (dead) labeling and CellTiter Glo viability assays were employed. In heterotypic LnCaP/PCAF spheroids, PI labeling revealed the formation of a necrotic core in both untreated and peptide‐treated spheroids. In PCa spheroids incubated with CAVPENET, dead cells were not limited to the internal region, but rather were throughout the spheroid, indicating that the peptide was inducing cell death. In the heterotypic spheroids incubated with CAVPENET control, the peptide‐induced cell death was attenuated (Figure 4A). The development of a necrotic core was also observed in PCAF spheroids, similar to what happened for heterotypic spheroids. Regarding the impact of peptides, cell death was seen throughout the entire spheroid area for both CAVPENET control and CAVPENET (Figure 4B).

At the concentration of 20 μM, while CAVPENET control did not significantly affect the viability of heterotypic LnCaP/PCAF 3D spheroids, contrariwise CAVPENET significantly decreased their viability (*p* = .0151). In fact, a significant difference was noted between the effect of both peptides at this concentration (*p* = .0372, Figure 4C). It is noteworthy here that CAVPENET significantly reduced the viability of LnCaP cells grown in 2D culture by ~50%.^13^ Interestingly, PCAFs were not significantly affected by 20 μM of either CAVPENET control or CAVPENET peptides (Figure 4D). At a higher concentration (30 μM) of both peptides, a decrease in the viability of cells in both LnCaP/PCAF and PCAF spheroids was observed (*p* <.0001) (Figure 4C,D).

### 3.4 Influence of CAVPENET on prostate cancer and stromal cells distribution

**FIGURE 5:**
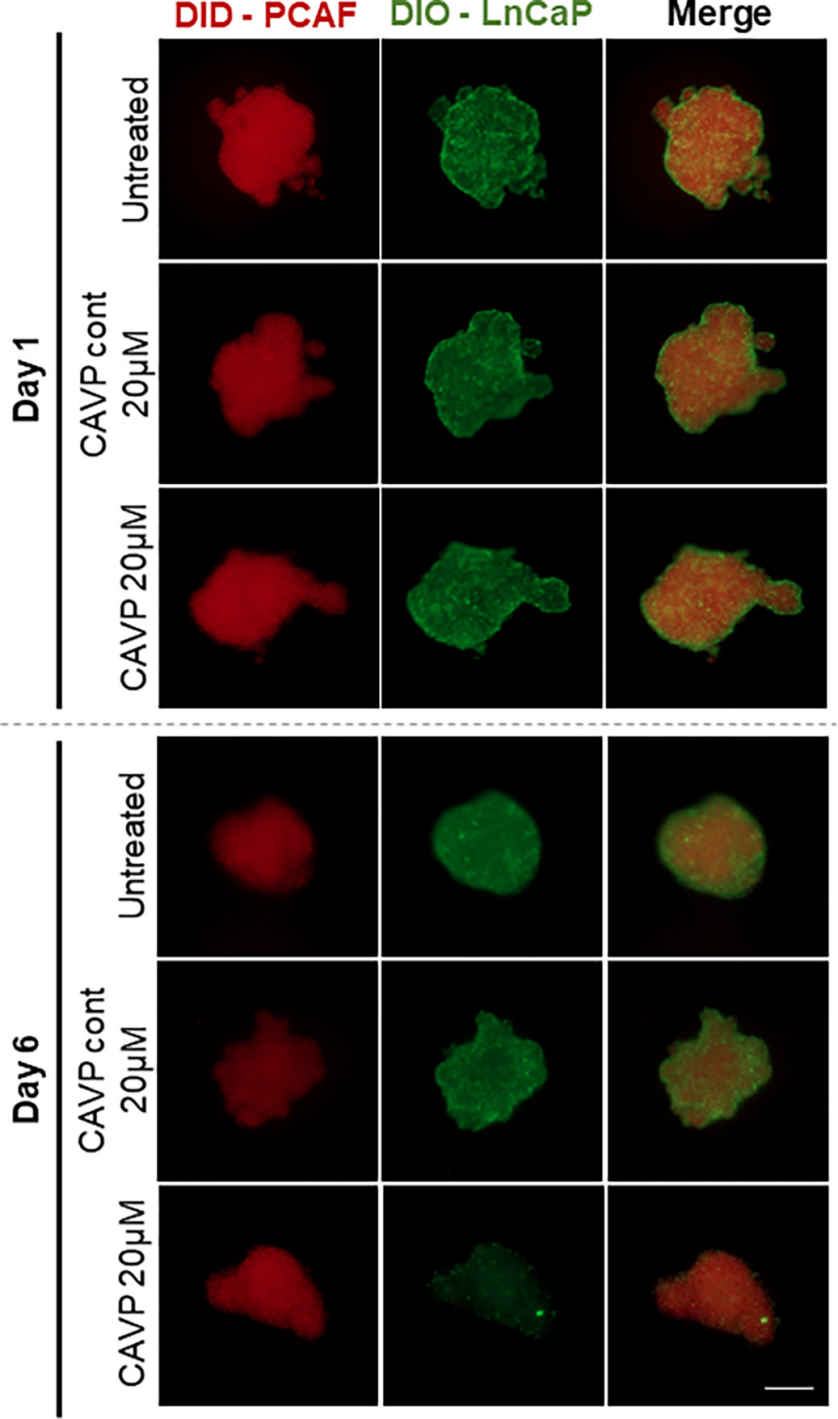
Cellular spatial organization in heterotypic LnCaP/PCAF 3D spheroids at day 1 and 6, after incubation with 20 μM of CAVPENET control and CAVPENET peptides. PCAF cells were labeled with DiD (red) and LnCaP cells with DiO (green). Scale bar: 200 μm.

Aiming to characterize the influence of CAVPENET control and CAVPENET peptides in the spatial distribution of cancer and stroma cells in the 3D heterotypic tumor spheroids, the LnCaP and PCAF cells were labeled with long term cell tracking dyes DiO and DiD, respectively. The cellular distribution of the two cell types was evaluated at day 1 and 6 (Figure 5). At day 1 (when the peptides were administered), the cellular organization of PCAF (red) and LnCaP cells (green) was similar for all the experimental conditions. As the cellular density increased, the PCAF cells were more surrounded by LnCaP cells in the untreated heterotypic spheroids (Figure 5). There were no significant alterations in the spatial distribution of the two cell types observed after treatment with CAVPENET control peptide. In contrast, in CAVPENET‐treated spheroids, the PCAF cells were less surrounded by LnCaP cells (Figure 5).

## 4 DISCUSSION

The ability of 3D spheroid models to recapitulate the *in vivo* organization and microenvironment of human tumors has been well documented.^23^, ^24^ Likewise, the significance of the tumor microenvironment, especially the active stroma, in PCa progression has been shown to impact resistance to candidate therapeutics.^25^ This underscores the relevance of employing heterotypic 3D models to study PCa in a preclinical setting moving forward from conventional and inherently limited flat monolayered cultures. Of relevance to this study, the incorporation of PCAFs into 3D PCa models enhances their ability to mimic the cellular elements present in the *in vivo* prostate tumors microenvironment.^26^

A previous work from our team highlighted the importance of tumor‐stroma prostate cancer *in vitro* models for therapeutic screening, providing a detailed morphological, histological and molecular characterization.^27^ Aiming to preclinically validate a candidate therapeutic peptide for PCa, here, we established a similarly simple and reproducible biomimetic 3D *in vitro* model of PCa, consisting of heterotypic 3D microtumor spheroids, comprising PCa cells and PCAFs, in which both cell types were randomly distributed across the microtumors' volume. For this model, LnCaP cells were selected as the PCa cell line based on the greater anticancer effect of CAVPENET in this cell type in 2D cultures and the documented association between AR and PP1/CAV1 interaction.^13^ Analyses of the diameters of spheroids within a 6‐day culture period revealed an increase in heterotypic spheroids' size, as a result of cellular proliferation. The PCAF cells are described as the main components of tumor stroma in PCa and are crucial to the development of self‐assembled compact and circular spheroids. This role is evidenced by the significant microtissue compaction of PCAF monoculture spheroids, which is primarily explained by strong cell–cell adhesions. Indeed, a noticeable reduction in the size of the PCAF 3D spheroids over time was observed. To further characterize the developed 3D PCa model, a live/dead cytotoxicity assay was performed. A clear necrotic core region was evident in both heterotypic LnCaP/PCAF and monotypic PCAF spheroids, with dead cells occupying the internal core of spheroids. The formation of a necrotic core is typical for PCa^24^, ^28^ and other cancer spheroids,^29^ as well as for some *in vivo* tumor tissues,^30^, ^31^ resulting from a deficient supply of nutrients and oxygen to the centre of the tumor.

The CAVPENET peptide exhibited clear anticancer activity in 2D monolayer PC‐3 and LnCaP cultures at a concentration range of 5–20 μM,^13^ underscoring the importance of evaluating its therapeutic potential in a more physiologically relevant model. The 3D spheroid models have been recognized as a mean to bridge the gap between 2D monolayer *in vitro* models and *in vivo* models in different aspects, including drug dosages.^32^ Considering the reported lower sensivity of 3D cancer models to diverse anticancer peptides,^33^ we evaluated the tumor‐suppressive effects of CAVPENET in the established 3D spheroid model at concentrations of 20 and 30 μM, to confirm its preclinical efficacy.

The capacity of cell‐penetrating peptides to be internalized in different types of cancer cells,^34^, ^35^ including PCa cells,^36^, ^37^ has been extensively documented. Nevertheless, the interaction and uptake of these peptides in 3D spheroids has remained highly unexplored so far, hence our aim to evaluate this aspect in such solid microtumor models. In this study, both CAVPENET peptide and a scrambled homologue (CAVPENET control) effectively interact with both monotypic and heterotypic 3D spheroids. This discovery aligns with the results reported by van den Brand et al. 2018,^38^ who described the interaction and penetration of cell‐penetrating peptides in cancer 3D spheroids.

The CAVPENET peptide was designed to target and disrupt the interaction between PP1 and CAV1, a protein–protein interaction described in PCa cells and with a reported role for the progression of this type of tumor.^12^ It is widely recognized that the formation of PP1 complexes highly depends on the tissue and the biological context,^39^, ^40^, ^41^ and there is no indication of PP1/CAV1 interaction occurring in fibroblasts. In fact, PP1 complexes have not been explored in CAF cells and even in the case of fibroblasts, there are few studies documented in the literature.^42^, ^43^, ^44^ Nevertheless, the CAFs CAV1 has been considered a potential drug target in cancer,^45^ and targeting this protein was associated with beneficial effects for PCa when combined with other therapies.^46^ In this study, although the CAVPENET peptide was able to interact with cells forming PCAF spheroids, their growth was not significantly affected at the concentration of 20 μM. Furthermore, despite an apparent increase in PI fluorescent signal in peptide‐treated spheroids, no significant alterations were observed in spheroids viability at the same concentration. These data suggest that CAVPENET does not directly affect PCAF cells when they are growing alone, likely because the biological target (PP1/CAV1 interaction) is absent in these cells. Notably, in preliminary experiments, monotypic PCa spheroids were also cultured; however, within the study timeframe, spheroids composed exclusively of PCa cells failed to form properly and became completely disorganized after 24 h of peptide treatment (Supplementary Figure 1). Based on these observations, we did not continue the analysis of peptides in this model.

Interestingly, CAVPENET treatment (20 μM) significantly reduced the growth and viability of cells in LnCaP/PCAF spheroids. Furthermore, there was a notable difference between the effect of CAVPENET and CAVPENET control at this concentration. In general, 3D cultures of PCa cells are more resistant to chemotherapeutic drugs compared to cancer cells cultured in 2D monolayers. The increased complexity of the 3D models, as well as the drug gradient formed in 3D spheroids, may explain this finding.^17^ Moreover, the addition of PCAFs to the PCa 3D models attenuated the death of PCa cells induced by several chemotherapeutic drugs.^21^ However, the impact of CAVPENET on 3D spheroid cells viability closely resembled the effect observed in LnCaP 2D cultures at the same concentration.^13^ The influence of CAFs on the signaling of cancer cells might, to some extent, explain this finding. Indeed, the activation of AKT pathway in cancer cells by CAFs, and its role in promoting cancer cells survival and proliferation has been documented in different types of cancer, including lung,^47^ and breast^48^ cancers. In PCa, although there is no direct evidence regarding the activation of AKT signaling in cancer cells by CAF cells, increased AKT phosphorylation and consequent activation was observed in the 3D LnCaP/CAF heterotypic model when compared to LnCaP 3D spheroids.^49^ This discovery may provide a rationale for the apparent anticancer effectiveness of CAVPENET in the 3D heterotypic tumor model, since AKT was regarded as the primary signaling pathway influenced by this peptide.^13^ Thus, while it appears that CAVPENET peptide predominantly targets LnCaP cells, the interplay between PCAF and cancer cells might be important for the anticancer activity of CAVPENET. The combination of several chemotherapeutic drugs with anti‐stromal drugs has been proposed. Indeed, combining gemcitabine with the anti‐stromal drug halofuginone strongly decreased the pancreatic cancer cells' viability when compared with the single agents.^50^ Thus, combining the CAVPENET peptide with an anti‐stromal drug may be a promising approach.

At the concentration of 30 μM, there was no discernible difference in the impact of peptides on both monotypic and heterotypic 3D spheroids. Interestingly, even in spheroids containing only PCAFs, both peptides strongly influenced spheroid growth and viability, indicating potential peptide‐induced toxicity due to an excessive concentration. The concentrations tested in this study align with doses examined for other bioactive cell‐penetrating peptides in 2D models and were considered physiological acceptable doses.^51^ Notably, CAVPENET peptide clearly retains its antitumoral activity in the 3D spheroid model at a dosage similar to that used in 2D PCa cultures, highlighting its preclinical efficacy. Moreover, these findings suggest that 20 μM could serve as a starting point for future *in vivo* studies of the CAVPENET peptide.

Overall, our research strengthens the suitability of employing 3D PCa‐stroma heterotypic spheroid models to evaluate the effectiveness of anticancer cell penetrating peptides in a preclinical setting. Moreover, our results confirm that the CAVPENET peptide retains its anti‐tumoral effect on PCa spheroids, which more closely mimics the *in vivo* conditions in comparison to conventional assays performed in 2D cell monolayers. These findings emphasize the potential of the CAVPENET peptide as an effective therapeutic approach for PCa, which warrants further investigation.

## AUTHOR CONTRIBUTIONS

**Bárbara Matos:** Conceptualization; data curation; formal analysis; investigation; methodology; writing – original draft; writing – review and editing. **Maria V. Monteiro:** Methodology; investigation; writing – review and editing. **Matilde R. Lagarto:** Methodology; investigation; writing – review and editing. **John Howl:** Conceptualization; supervision; writing – review and editing. **Carmen Jerónimo:** Supervision; writing – review and editing. **Vítor M. Gaspar:** Conceptualization; funding acquisition; writing – review and editing. **João F. Mano:** Conceptualization; funding acquisition; writing – review and editing. **Margarida Fardilha:** Conceptualization; funding acquisition; supervision; writing – review and editing.

## FUNDING INFORMATION

This work was developed within the scope of the project CICECO‐Aveiro Institute of Materials, UIDB/50011/2020, UIDP/50011/2020 & LA/P/0006/2020, financed by national funds through the FCT/MEC (PIDDAC), and iBiMED (UIDB/04501/2020), funded by FCT. The authors acknowledge the financial support by the Portuguese Foundation for Science and Technology (FCT) through Doctoral Grants (SFRH/BD/146032/2019, Bárbara Matos, and DFA/BD/7692/2020, Maria V. Monteiro). The authors also acknowledge the financial support by the Portuguese Foundation for Science and Technology (FCT) through an Assistant Researcher contract (CEECIN/02106/2022, V.M.G.).

## CONFLICT OF INTEREST STATEMENT

The authors declare no conflict of interest.

## Supporting information


**Supplementary Figure 1:** PCa cell monotypic 3D spheroids after 24 h incubation with 20 and 30 μM of CAVPENET control and CAVPENET peptides.

## Data Availability

The data that support the findings of this study are available from the corresponding author upon reasonable request.
