# Supplementary material for: Screening anticancer peptides performance in organotypic prostate tumor‐stroma 3D models
Source: Int J Cancer. 2026 Jan 13;158(11):2936–46. doi: 10.1002/ijc.70333 (PMC13047245; doi:10.1002/ijc.70333)
Supplement: Supplementary file 1 — Supplementary Figure 1: PCa cell monotypic 3D spheroids after 24 h incubation with 20 and 30 μM of CAVPENET control and CAVPENET peptides. [file IJC-158-2936-s001.pdf]

Supplementary Material for

Screening Anticancer Peptides Performance in Organotypic Prostate Tumor-Stroma 3D Models

Bárbara Matos, Maria V. Monteiro, Matilde R. Lagarto, John Howl, Carmen Jerónimo, Vítor M. Gaspar, João F. Mano, Margarida Fardilha

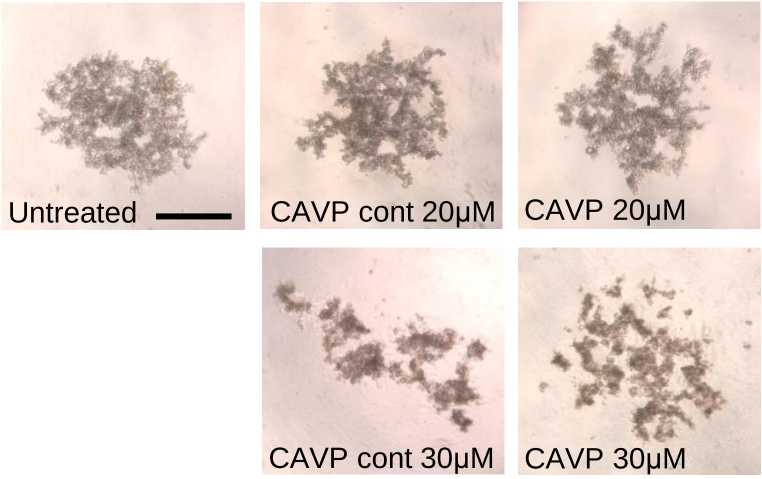

Supplementary Figure 1: PCa cell monotypic 3D spheroids after 24h incubation with 20 and 30 μM of CAVPENET control and CAVPENET peptides. A representative image of each condition is represented (Scale bar: 300μm).
